# Hypertension diagnosis, awareness, treatment, and control in Sri Lankan adults: a nationally representative cross-sectional study

**DOI:** 10.1186/s12889-025-22659-7

**Published:** 2025-04-24

**Authors:** Ravindra P. Rannan-Eliya, Nilmini Wijemunige, Bilesha Perera, Prasadini Perera, H. M. M. Herath, Vajira H. W. Dissanayake, SLHAS Collaborators, Shanti Dalpatadu, Sarath Samarage, Anuji Gamage

**Affiliations:** 1 Institute for Health Policy, 72 Park Street, Colombo, 00200 Sri Lanka; 2 University of Ruhuna, Galle, Sri Lanka; 3 University of Peradeniya, Peradeniya, Sri Lanka; 4 University of Colombo, Colombo, Sri Lanka

**Keywords:** Hypertension, Sri Lanka, Care cascade, Diagnosis, Treatment, Control, Health equity

## Abstract

**Background:**

Sri Lanka’s health policies prioritize improving hypertension control. The care cascade framework, a valuable tool that has informed hypertension control strategy in many countries, has not been assessed previously in Sri Lanka. This study addresses this gap using data from a nationally representative, longitudinal cohort study, providing a baseline for future monitoring.

**Methods:**

We analysed data from the 2018–2019 first wave of the Sri Lanka Health and Ageing Study (SLHAS). The extended care cascade included: (i) prevalence, (ii) ever screened, (iii) diagnosis, (iv) awareness, (v) treatment, (vi) adherence, and (vii) control. We used multivariate logistic regression to assess factors associated with attainment of each step, conditional on prevalence and on treatment, and we assessed socioeconomic inequalities using concentration indices. We also compared performance against global estimates, and against regional and middle-income countries.

**Results:**

We included 6,342 participants in the analysis. The weighted prevalence of hypertension was 27.6%. Of those with hypertension, 87% had their blood pressure (BP) ever measured; 53% were diagnosed; 50% were aware of their condition; 44% were taking treatment; and 20% had their blood pressure under control. Females and older adults had better cascade outcomes, but this was driven by higher rates of diagnosis. Diabetes and increased body mass index (BMI) were associated with higher diagnosis rates, but not treatment and control after diagnosis. Education and sector of residence were not associated with disparities, but treatment rates were higher in the most socioeconomic advantaged. Concentration indices confirmed pro-rich inequality across the care cascade from ever screened to treatment and adherence, but control was not unequal, with the data suggesting better control by public providers, whom the poor relied on more.

**Conclusions:**

Only one in five Sri Lankans with hypertension achieve BP control, with the biggest cascade losses occurring at diagnosis and control after treatment. Our findings point to a substantial influence of providers. Efforts to improve hypertension control in Sri Lanka should focus on increasing detection through opportunistic screening and improving interventions to improve physician control of BP. These findings provide actionable insights for further research and to strengthen hypertension control efforts in Sri Lanka.

**Supplementary Information:**

The online version contains supplementary material available at 10.1186/s12889-025-22659-7.

## Background

Hypertension is a leading cause of cardiovascular disease (CVD) and chronic kidney disease (CKD) worldwide. Effective interventions exist that can lower blood pressure both at the population and individual level. The 2030 Sustainable Development Goals set specific targets to lower blood pressure and to increase treatment coverage, yet substantial global gaps remain in detection of those at risk and in coverage by effective treatment. These are higher in low and middle-income countries (LMICs) than in high-income countries (HICs) [1].

Sri Lanka, a South Asian LMIC, is experiencing rapid transformations associated with increased risk of hypertension, including ageing, and increased salt intake, affluence, physical inactivity, and urbanization [2, 3]. By 2019, hypertension prevalence in Sri Lankan adults was 28%[4] under the traditional JNC7 (seventh Joint National Committee report) definition [5], and 51% under the more recent and stricter ACC/AHA (American College of Cardiology/American Heart Association) 2017 guidelines [6]. Recognizing the importance, health authorities have prioritizing improving hypertension care as a national priority, and they have invested in new services to screen for high blood pressure, and in strengthening healthcare facilities to better manage hypertension.

Globally, regular assessment of the hypertension care cascade has been a key tool in improving hypertension management, with the United States being an influential example [7–9]. In Sri Lanka, several local studies have highlighted gaps in hypertension diagnosis, treatment and control [10, 11], but no comprehensive national assessments of the care cascade have been conducted. There have also been no attempts to benchmark performance with other countries, nor any initiatives to track changes at the population level. Identifying shortfalls in hypertension care and understanding factors driving disparities are critical for evaluating the current interventions, and guiding future priorities.

This study addresses these gaps by analysing baseline data from a national longitudinal health survey to: (1) quantify attainment of each step in the hypertension care cascade; (2) assess population disparities at each step; (3) identify individual, area and provider-level correlates of underperformance; and (4) establish a national baseline to track future progress in Sri Lanka.

## Methods

### Data source

We used data from the first wave of the Sri Lanka Health and Ageing Study (SLHAS), which was conducted from mid-November 2018 to mid-November 2019. The SLHAS is a national, longitudinal cohort study approved by the Ministry of Health (MOH), Sri Lanka. It used a stratified, multistage probability sampling design to recruit a representative sample (after reweighting) of the non-institutionalized adult population of Sri Lanka [4]. Stratification was by district, sector of residence (urban, rural, estate, rural/estate), and quantile group of an index of area socioeconomic status (SES). This SES index was derived using principal components analysis (PCA) of over one hundred small area indicators obtained from the 2012 national population census [4, 12].

Within strata, grama niladhari divisions (GNDs) (*N* = 14,104) – the smallest administrative unit in Sri Lanka – were selected using probability proportionate to the size of their adult population. Households within each primary sample unit (PSU) were then selected by systematic sampling from random starting points. Selected households received an official letter from MOH encouraging participation. Within each household, one adult was randomly selected, with age-sex weighting, to be invited to participate in the study. Eligible adults were individuals aged 18 years or over residing at the household, non-pregnant, and able to provide informed consent. If consent was declined, another household was selected.

Consenting individuals attended a nearby field clinic, typically the premises of a MOH clinic that were not being used that day, where they were interviewed, had anthropometrics and blood pressure measured, and gave blood and urine samples. Individuals with mobility limitations were interviewed at home with a shorter examination that included blood pressure.

### Measurements

Trained field staff, comprising medical or nursing graduates, took two blood pressure readings, 10 min apart, using an OMRON HEM-7320 Automatic BP Monitor (Omron Corp., Kyoto, Japan) [4]. All devices were validated for accuracy against a mercury column sphygmomanometer. Measurements were taken after participants rested for 10 min in a sitting position, with the arm resting at heart level. The right arm was used unless contraindicated.

Height was measured to the nearest 0.1 cm using a Seca 240 cm height measure (Seca GmbH, Hamburg, Germany). Weight was measured to the nearest 0.1 kg using an OMRON BF511 Body Composition Monitor. The body mass index (BMI) was calculated as weight (kg) divided by the square of height (m).

Participants were asked to bring personal medical records and any medicines they were taking to the interview. In Sri Lanka, patients being treated for chronic conditions are typically given treatment record books. Interviewers asked participants if they had been previously diagnosed with hypertension, and checked their medical records, if available, for any such diagnosis. They recorded any medicines taken in the past 14 days, and these were coded to the WHO Anatomical Therapeutic Chemical (ATC) classification. Interviewers also collected information on participants’ demographic characteristics, housing conditions, sanitation, water supply, and household assets.

### Definitions of hypertensions and the care cascade

We defined a participant as having hypertension if (a) they had a systolic blood pressure (SBP) ≥ 140 mmHg, or a diastolic blood pressure (DBP) ≥ 90 mmHg, or (b) they took any antihypertensive medication and either reported or had evidence in their medical records of a previous diagnosis of hypertension, or (c) took any antihypertensive medication and reported hypertension as the reason for taking that medication. SBP and DBP was each defined as the average of the two measurements. Antihypertensive medications were defined as medicines in ATC classes C02CA01, C03A, C07–C08, C09 A–C09D, C10BX03–C10BX04, C10BX06–C10BX07, C10BX09–C10BX15, and C09C–C09D.

Our blood pressure thresholds align with the JNC7 definition [5], rather than the ACC/AHA 2017 guidelines [6]. This choice reflects the current situation in Sri Lanka where hypertension treatment guidelines and most clinical practice uses the older JNC7 thresholds. Additionally, the hypertension care cascade literature to date almost exclusively uses the JNC7 definitions. Our definition is slightly narrower than that used in a previous study that used SLHAS data to estimate hypertension prevalence[4] in that we only classified individuals taking antihypertensives as having hypertension if there is evidence of a diagnosis of hypertension or if they report they are taking medicines for hypertension. This recognizes that antihypertensive medications are sometimes prescribed for reasons other than hypertension, and it aligns our definition with those used in other hypertension cascade studies [13, 14].

We examined the following steps in an extended hypertension care cascade[7, 15]: ever screened, diagnosed, aware, treated, adherent, and controlled. We defined a participant as: (1) *ever screened* if they reported ever having had their blood pressure measured; (2) *diagnosed* if they had hypertension and either they reported having been diagnosed or such a diagnosis was noted in their medical records; (3) *aware* if they had hypertension and they reported having been diagnosed with hypertension; (4) *treated* if they had hypertension and were taking antihypertension medications; (5) *adherent* to medication if they had hypertension and they reported taking antihypertension medicines at least once every day for the past 14 days; and (5) *controlled* if they had hypertension and they had SBP < 140 mmHg and DBP < 90 mmHg.

Our definition of treatment is limited to pharmacological therapy, as the SLHAS did not collect information on non-pharmacological therapies, such as salt or diet restriction. Participants reporting treatment for hypertension were classified by treatment source as *public* (using only government providers), *private* (using only private providers)*,* or *mixed* (using both government and private providers for regular hypertension care).

We defined a participant as having diagnosed diabetes if they reported a prior diagnosis of diabetes. Household SES was measured using an index derived from PCA of indicators such as housing materials, sanitation, water supply, and possession of selected assets [12, 16]. Participants were grouped into SES quintile groups, based on this index. We classified PSUs into area SES tertiles using the same index applied for sample stratification.

### Statistical analyses

We selected participants who met our criteria for having hypertension and estimated the percentage at each step in the care cascade overall and by sociodemographic characteristics (sex, age group, ethnicity, education, sector, household SES), area SES, province, diagnosis of diabetes, and source of hypertension care. We stratified by diabetes diagnosis because standard treatment guidelines in Sri Lanka require that diabetes patients be assessed and treated for hypertension [17], and there is evidence that having comorbidities influences hypertension control [18].

We used a concentration index—the scaled covariance between an outcome and rank in the distribution of the household SES index—to quantify the degree of inequality by SES at each step of the care cascade [19]. We used indirect standardization to adjust this measure of inequality for differences in age and sex [20], by using 5-year age-sex groups as controls. A positive (negative) concentration index indicates higher rates of the outcome–treated, for example–among richer (poorer) individuals. We also used concentration indices to measure socioeconomic-related inequalities in use of public and private providers as the source of routine hypertension care.

We used hierarchical multivariate logistic regression models to identify covariates influencing different care cascade steps: ever screened and diagnosed conditional on being hypertensive; aware and treated conditional on being diagnosed; and adherent and controlled conditional on being treated. For these analyses, we did not apply sample weights because the model regressors included the characteristics that were used to construct the weights [21].

For all other analyses, we applied sampling weights and accounted for the clustered sampling design with a finite population correction and estimating variances using Taylor linearization. We obtained sample weights using iterative proportional fitting (IPF) to adjust the data’s initial post-stratification design weights. This matches the sample to the national population (2012 census) by age, sex, province, socioeconomic status, sector, and ethnicity, as previously described [4, 16].

All analyses were performed using statistical software Stata version 17.0 (StataCorp, College Station, Texas, USA).

## Results

### Sample characteristics

The SLHAS Wave 1 survey had an effective response rate of 65.0% of households that were contacted, with 6,668 individuals recruited from 298 PSUs within 117 strata, of whom 41 were interviewed and examined at home. The final sample was well-balanced by sex, age, sector, socioeconomic status, and ethnicity. We excluded 3 SLHAS participants aged < 18 years, and 323 with missing data for taking antihypertensive medication or lacking either SBP or DBP measurements, leaving 6,342 (95.1%) participants for analysis from 298 PSUs. This sample is identical to that used for estimating hypertension prevalence, as previously published [4]. Their mean age (± SD) was 49.9 (± 17.2), and 51.0% (3,235) were female, and 16.7% (1,057) self-reported a diagnosis of diabetes. The sample had underrepresentation of younger adults and Muslims, but after weighting matched the national population on age, sex, ethnicity, and area and household SES (Supplementary Table 1).

Within the overall sample, 2,218 individuals had hypertension, equivalent with weighting to a prevalence rate of 27.6%. This is lower than the previously published estimate of hypertension prevalence using the same sample (28.2%) [4], as we used a narrower definition of hypertension as explained above. The subsample with hypertension is older, more urbanized, and more affluent than the overall population. The weighted prevalence of self-reported diabetes (26.3%) in adults with hypertension is double that of all adults (13.9%), which is expected as diabetes and hypertension are frequent comorbidities.

### Hypertension care cascade

**Fig. 1:**
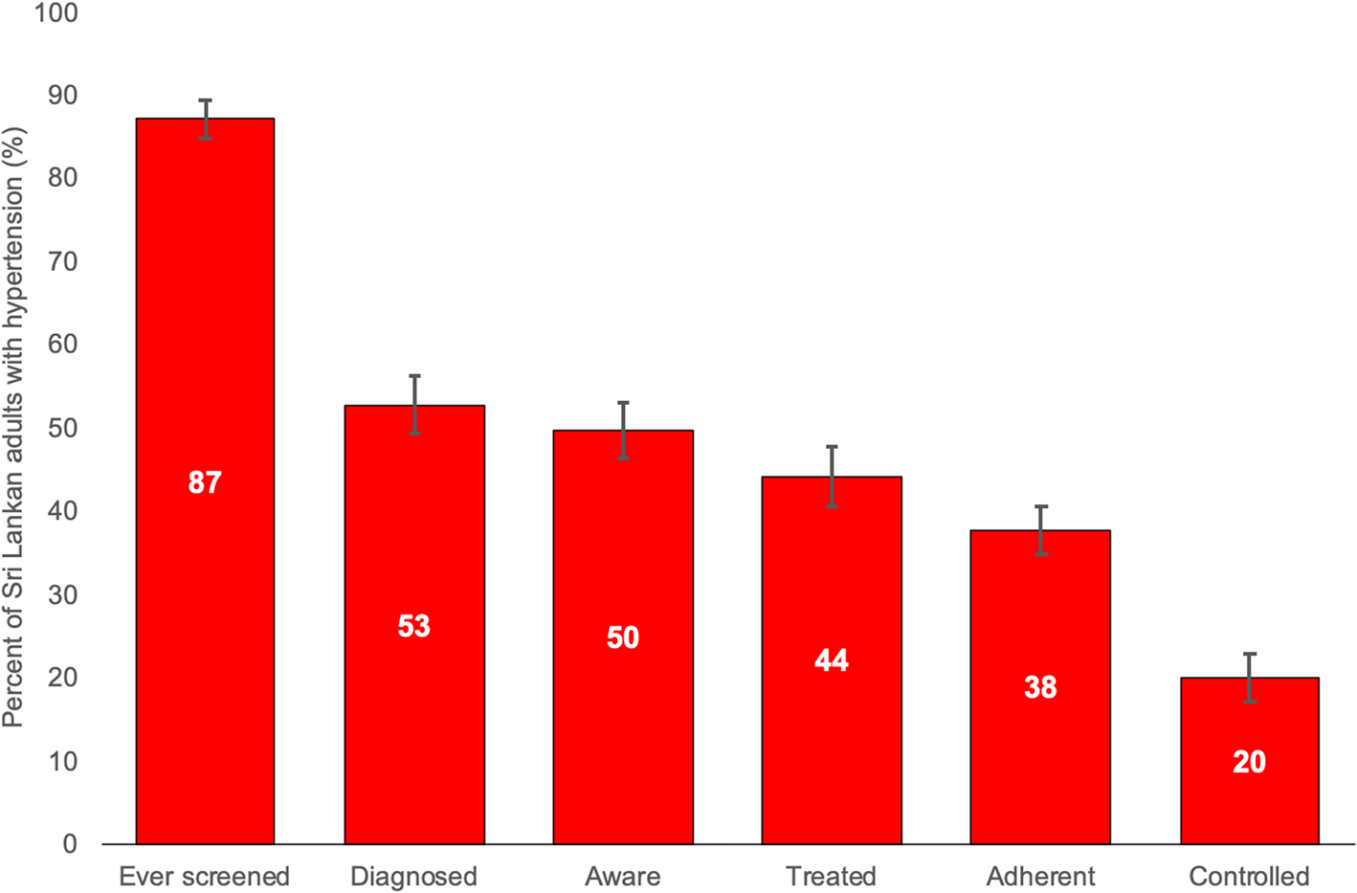
Cascade of hypertension care in Sri Lankan adults aged ≥ 18 years, SLHAS 2018/2019 estimates. *Notes:* Bars with 95% confidence intervals

Table 1 and Table 2 provide weighted estimates of the major care cascade steps, overall and by population characteristics, with confidence intervals (CIs). In Sri Lankan adults with hypertension in 2019: (i) 87.1% (95% CI 84.9–89.3) had been ever screened; (ii) 52.8% (95% CI 49.3–56.2) were diagnosed; (iii) 49.6% (95% CI 46.2–53.0) were aware of their condition; (iv) 44.2% (95% CI 40.7–47.7) were being treated; (v) 37.8% (95% CI 34.8–40.7) were adherent; and (vi) 20.0% (95% CI 17.1–22.8) were controlled (Fig. 1).

**Table 1:** Prevalence of being ever screened, diagnosed, aware, and treated among Sri Lankan adults with hypertension

	Ever screened	Diagnosed	Aware	Treated
	% (95% CI) N = 2,205	% (95% CI) N = 2,218	% (95% CI) N = 2,218	% (95% CI) N = 2,218
Overall	87.1 (84.9–89.3)	52.8 (49.3–56.2)	49.6 (46.2–53.0)	44.2 (40.7–47.7)
Sex				
Male	82.7 (79.1–86.3)	43.5 (39.4–47.6)	40.2 (36.4–44.1)	36.0 (32.0–40.1)
Female	90.9 (88.3–93.6)	60.9 (56.2–65.7)	57.9 (53.2–62.6)	51.4 (46.3–56.4)
Age (years)				
18–29	77.8 (66.1–89.5)	0.4 (− 0.4–1.2)	0.4 (− 0.4–1.2)	0.0^a^
30–39	74.8 (67.3–82.4)	26.5 (17.1–36.0)	26.2 (16.8–35.6)	17.9 (9.5–26.4)
40–49	83.1 (77.6–88.7)	37.3 (29.9–44.8)	36.0 (28.6–43.5)	26.7 (20.6–32.8)
50–59	90.0 (86.4–93.6)	58.8 (51.5–66.0)	56.3 (49.0–63.7)	48.3 (41.6–55.1)
60–69	89.9 (86.3–93.5)	65.1 (59.4–70.8)	59.2 (53.6–64.8)	57.7 (51.8–63.6)
70–79	92.3 (88.7–96.0)	77.0 (72.8–81.2)	71.9 (67.1–76.7)	67.0 (62.6–71.4)
80 +	96.5 (92.8–100.3)	68.5 (52.3–84.7)	64.1 (47.9–80.4)	64.5 (50.3–78.7)
Ethnicity				
Sinhala	89.3 (86.8–91.8)	51.5 (47.5–55.5)	48.3 (44.5–52.1)	44.4 (40.3–48.5)
SL Tamil	82.9 (78.5–87.2)	49.1 (41.8–56.4)	44.8 (38.0–51.6)	40.1 (32.7–47.5)
Indian Tamil	59.6 (42.1–77.1)	42.9 (26.5–59.3)	42.0 (25.8–58.2)	30.5 (14.2–46.7)
Muslim	85.2 (78.3–92.2)	67.1 (56.0–78.2)	65.1 (53.9–76.3)	50.9 (38.2–63.5)
Sector				
Urban	88.6 (85.8–91.4)	62.2 (57.1–67.3)	57.9 (53.0–62.7)	54.3 (48.9–59.7)
Rural	88.1 (85.3–90.9)	50.4 (46.0–54.8)	47.7 (43.4–52.0)	41.6 (37.1–46.0)
Estate	73.3 (65.9–80.6)	46.2 (34.9–57.5)	41.3 (31.5–51.1)	32.3 (22.4–42.2)
Rural/Estate	75.4 (64.3–86.6)	44.4 (32.0–56.8)	41.0 (28.6–53.4)	36.4 (25.8–47.0)
Education				
No schooling	90.4 (85.1–95.7)	67.6 (55.8–79.5)	65.3 (53.2–77.3)	57.4 (44.4–70.4)
Primary	84.7 (79.4–89.9)	60.1 (52.9–67.2)	55.5 (48.5–62.6)	50.3 (43.1–57.6)
Secondary	87.2 (84.7–89.7)	50.4 (46.8–54.0)	47.4 (44.0–50.8)	42.5 (38.9–46.1)
Tertiary	93.0 (84.7–101.4)	54.1 (40.3–68.0)	52.5 (38.9–66.2)	38.7 (21.4–56.1)
Household SES quintile				
Poorest	84.1 (79.3–88.8)	49.4 (42.4–56.3)	46.5 (39.5–53.6)	41.4 (34.0–48.8)
2	82.9 (77.6–88.3)	56.4 (49.5–63.4)	52.7 (45.9–59.5)	47.8 (41.2–54.3)
3	88.0 (83.9–92.2)	49.2 (43.1–55.2)	46.5 (40.5–52.4)	35.3 (29.3–41.3)
4	89.6 (86.0–93.2)	53.6 (47.2–60.0)	50.4 (44.1–56.6)	48.7 (42.4–55.0)
Wealthiest	89.9 (85.8–94.1)	55.1 (48.9–61.2)	51.8 (45.6–57.9)	47.1 (41.1–53.1)
Area SES tertile				
Least developed	81.3 (76.8–85.9)	41.2 (35.9–46.5)	38.2 (33.2–43.1)	31.8 (25.9–37.7)
Middle	91.1 (87.9–94.2)	53.9 (47.3–60.6)	51.3 (44.5–58.0)	46.0 (39.4–52.5)
Most developed	88.1 (85.2–91.0)	59.9 (54.8–65.1)	56.3 (51.5–61.2)	51.4 (46.3–56.6)
Diabetes (self-reported)				
No	84.0 (81.1–86.9)	44.3 (40.2–48.3)	41.4 (37.5–45.4)	36.4 (32.6–40.3)
Yes	95.8 (93.5–98.1)	76.5 (71.8–81.2)	72.5 (67.7–77.3)	65.8 (60.4–71.2)
Province				
Western	90.6 (86.6–94.6)	56.7 (50.0–63.5)	53.0 (46.9–59.1)	49.5 (42.8–56.1)
Central	85.7 (79.1–92.2)	52.0 (43.2–60.8)	49.7 (40.9–58.6)	42.3 (30.9–53.7)
Southern	88.9 (84.5–93.2)	51.7 (44.0–59.4)	49.4 (41.9–57.0)	43.8 (37.9–49.7)
Northern	83.7 (77.0–90.3)	54.1 (46.4–61.7)	50.2 (41.8–58.7)	48.6 (41.7–55.4)
Eastern	83.0 (74.5–91.4)	46.6 (33.1–60.1)	44.8 (31.3–58.3)	31.1 (20.7–41.6)
North-Western	88.9 (83.4–94.5)	53.7 (44.0–63.5)	50.8 (40.3–61.2)	44.5 (36.4–52.5)
North-Central	86.4 (80.4–92.3)	47.5 (39.3–55.8)	45.5 (37.7–53.3)	40.6 (32.3–48.9)
Uva	78.6 (70.5–86.7)	43.4 (27.9–58.9)	39.4 (26.2–52.6)	37.8 (24.1–51.5)
Sabaragamuwa	81.0 (72.4–89.6)	49.6 (36.8–62.3)	45.7 (31.4–59.9)	37.1 (24.6–49.5)

^a^Insufficient sample to provide estimates of confidence intervals

**Table 2:** Prevalence of being adherent or controlled among Sri Lankan adults with hypertension, all and treated

	Adherent	Controlled	Adherent	Controlled
	% (95% CI)	% (95% CI)	% (95% CI)	% (95% CI)
Conditional on	Having hypertension *N* = 2,218		Being treated *N* = 1,113	
Overall	37.8 (34.8–40.7)	20.0 (17.1–22.8)	85.5 (82.3–88.6)	45.2 (40.5–49.8)
Sex				
Male	30.8 (27.0–34.5)	14.7 (11.8–17.7)	85.4 (80.3–90.6)	40.9 (35.1–46.7)
Female	43.9 (39.7–48.1)	24.5 (20.9–28.2)	85.5 (81.8–89.2)	47.8 (42.1–53.5)
Age (years)				
18–29	0.0^a^	0.0^a^	^b^	^b^
30–39	16.0 (7.7–24.3)	6.8 (1.8–11.8)	88.9 (76.6–101.3)	37.9 (13.6–62.2)
40–49	20.2 (14.4–26.0)	12.1 (7.3–17.0)	75.6 (64.4–86.7)	45.5 (31.7–59.3)
50–59	41.3 (35.4–47.2)	21.1 (17.0–25.3)	85.4 (79.7–91.2)	43.8 (37.3–50.2)
60–69	49.0 (43.2–54.8)	28.6 (23.6–33.7)	84.9 (79.5–90.2)	49.6 (42.7–56.5)
70–79	59.1 (52.7–65.4)	28.2 (23.0–33.3)	88.1 (80.7–95.5)	42.0 (35.0–49.1)
80 +	59.2 (45.5–72.9)	29.0 (17.8–40.3)	91.8 (83.4–100.2)	45.0 (31.7–58.3)
Ethnicity				
Sinhala	39.0 (35.6–42.4)	22.0 (18.6–25.4)	87.9 (84.1–91.6)	49.6 (44.8–54.4)
SL Tamil	32.9 (25.6–40.3)	18.3 (13.1–23.4)	82.1 (76.1–88.1)	45.6 (35.7–55.5)
Indian Tamil	26.3 (8.2–44.3)	10.7 (− 1.3–22.7)	86.2 (65.9–106.5)	35.2 (5.6–64.7)
Muslim	37.9 (25.8–50.1)	10.9 (6.2–15.7)	74.6 (64.4–84.7)	21.5 (12.0–30.9)
Sector				
Urban	43.7 (38.6–48.8)	22.4 (18.1–26.7)	80.5 (74.4–86.6)	41.3 (34.8–47.8)
Rural	36.4 (32.7–40.1)	20.0 (16.2–23.7)	87.5 (83.4–91.6)	48.0 (41.5–54.5)
Estate	25.1 (17.2–33.1)	10.0 (5.3–14.8)	77.8 (75.4–80.3)	31.0 (18.2–43.9)
Rural/Estate	32.4 (21.8–43.0)	13.4 (6.2–20.7)	89.0 (82.4–95.6)	36.9 (22.1–51.8)
Education				
No schooling	47.7 (34.7–60.7)	33.0 (21.0–45.0)	83.1 (68.1–98.1)	57.5 (39.0–76.0)
Primary	43.6 (36.3–50.9)	26.0 (20.1–31.9)	86.6 (79.8–93.4)	51.6 (43.2–60.1)
Secondary	36.2 (33.1–39.3)	18.1 (15.2–21.0)	85.2 (81.3–89.2)	42.6 (37.3–47.9)
Tertiary	34.4 (17.3–51.4)	18.0 (3.3–32.6)	88.8 (75.8–101.9)	46.4 (21.0–71.8)
Household SES quintile				
Poorest	37.3 (30.3–44.3)	22.0 (16.1–27.8)	90.1 (85.2–94.9)	53.0 (43.3–62.7)
2	41.4 (35.6–47.1)	20.7 (15.8–25.5)	86.6 (80.7–92.5)	43.3 (35.6–50.9)
3	28.1 (22.3–33.9)	17.3 (11.5–23.1)	79.6 (71.1–88.1)	49.1 (37.5–60.7)
4	40.6 (34.5–46.6)	21.1 (15.8–26.4)	83.3 (76.0–90.5)	43.2 (34.3–52.2)
Wealthiest	41.0 (35.0–47.1)	18.9 (14.9–22.9)	87.1 (81.8–92.5)	40.1 (32.4–47.8)
Area SES tertile				
Least developed	28.2 (23.2–33.2)	15.8 (11.6–20.1)	88.6 (84.1–93.1)	49.7 (42.1–57.3)
2	38.7 (32.7–44.7)	21.9 (16.1–27.7)	84.2 (78.7–89.6)	47.6 (37.2–58.0)
Most developed	43.7 (39.7–47.7)	21.4 (17.4–25.3)	85.0 (79.8–90.1)	41.6 (36.1–47.1)
Diabetes (self-reported)				
No	31.2 (27.9–34.4)	17.3 (14.2–20.4)	85.5 (81.5–89.4)	47.5 (41.8–53.2)
Yes	56.3 (51.1–61.5)	27.4 (22.6–32.1)	85.4 (79.5–91.4)	41.6 (34.7–48.4)
Province				
Western	40.8 (35.8–45.8)	22.3 (16.3–28.3)	82.5 (76.6–88.3)	45.1 (37.2–53.0)
Central	35.4 (25.2–45.7)	14.1 (6.7–21.6)	83.7 (76.5–90.8)	33.4 (16.5–50.3)
Southern	39.6 (33.1–46.2)	20.6 (16.5–24.6)	90.5 (86.6–94.3)	46.9 (40.3–53.6)
Northern	39.8 (32.8–46.8)	25.2 (15.3–35.0)	82.0 (76.3–87.6)	51.9 (35.6–68.1)
Eastern	27.3 (17.4–37.2)	13.6 (7.8–19.3)	87.8 (80.2–95.3)	43.6 (25.5–61.6)
North-Western	39.3 (29.8–48.8)	21.5 (14.3–28.7)	88.3 (79.3–97.4)	48.4 (37.0–59.8)
North-Central	37.1 (29.5–44.8)	22.8 (13.9–31.7)	91.5 (85.0–98.1)	56.2 (41.1–71.3)
Uva	34.1 (21.4–46.8)	16.5 (7.3–25.7)	90.2 (76.8–103.6)	43.6 (30.7–56.6)
Sabaragamuwa	32.7 (22.6–42.9)	18.5 (10.1–26.9)	88.3 (73.2–103.5)	49.9 (36.8–63.1)

^a^Insufficient sample to provide estimates of confidence intervals, ^b^No observations

Females had better outcomes than men at all steps of the care cascade from screening and diagnosis to control. Attainment generally improved with age, except for control which plateaued at 28–29% in adults aged 60 years and more. All cascade steps were lower in Indian Tamils and estate areas. While screening, diagnosis, and treatment were higher among Muslims, control was lower. More educated adults were more likely to have ever been screened, but less likely to be diagnosed, under treatment, or achieve control. The tabulations also suggest the possibility of a pro-rich gradient in treatment, but a pro-poor gradient in control. Attainment varied by province, with control rates ranging from 14% in Central and Eastern provinces to 25% in Northern Province.

Of those currently been treated for hypertension with medication, 85.5% (95% CI 82.3–88.6) were adherent to medication, and 45.2% (95% CI 40.5–49.8) were controlled (Table 2). There were no large disparities for adherence, although it was only 78% in estate areas. In contrast there were more apparent disparities in control. This was higher in females (48%), the poorest quintile of households (53%), and those living in the least developed tertile of areas (50%), and lower in Indian Tamils (35%) and Muslims (21%).

### Hierarchical logistic regression models

The main adjusted odds ratio (aOR) results from the logistical regression models are presented in Table 3 (complete results given in Supplementary Tables 2–4). All models control for a wide range of both individual, household and community level variables, including age, sex, ethnicity, education, socioeconomic status, and province. The models for adherence and control also control for the source of regular hypertension care.

**Table 3:** Adjusted odds ratios for attaining each care cascade step in Sri Lankan adults with hypertension

	**Ever screened**	**Diagnosed**	**Aware**	**Treated**	**Adherent**	**Controlled**
**Conditional on**	Having hypertension		Being diagnosed		Being treated	
**Sex**						
Male	1.00	1.00	1.00	1.00	1.00	1.00
Female	1.92***	1.72***	1.04	0.91	0.86	1.23
**Age (years)**						
18–29	0.47*	0.03**	0.10	1.00		
30–39	0.63	0.45**	0.95	0.61	0.85	0.72
40–49	1.00	1.00	1.00	1.00	1.00	1.00
50–59	1.50	2.27***	1.32	1.85*	1.59	0.83
60–69	1.96**	3.14***	1.09	2.36***	1.42	0.81
70–79	2.54***	6.71***	1.77*	3.03***	2.03*	0.60
80 +	4.81**	7.84***	1.74	3.64***	2.69	0.74
**Ethnicity**						
Sinhala	1.00	1.00	1.00	1.00	1.00	1.00
SL Tamil	0.41***	0.89	1.04	0.79	0.36**	0.50*
Indian Tamil	0.22***	0.97	2.30	0.50	0.42	0.27**
Muslim	0.49*	1.59	1.31	0.61	0.48*	0.27***
**SES quintile**						
Poorest	1.00	1.00	1.00	1.00	1.00	1.00
2	0.97	1.19	1.09	0.94	1.00	0.83
3	1.48	1.06	0.82	0.78	0.87	0.96
4	1.74*	1.12	1.09	1.53	0.89	1.08
Wealthiest	1.67*	1.10	0.93	1.21	1.60	0.94
**Area SES tertile**						
Least developed	1.00	1.00	1.00	1.00	1.00	1.00
2	1.20	0.99	1.46	1.93**	0.77	0.93
Most developed	0.83	1.11	1.68	2.13**	1.43	0.86
**Diabetes (self-reported)**	3.14***	3.18***	1.02	1.10	1.19	0.91
**WHO BMI categories**						
Underweight (< 18.5)	1.00	1.00	1.00	1.00	1.00	1.00
Normal (18.5–24.9)	1.04	1.66*	1.87*	1.25	0.95	1.37
Overweight (25–29.9)	1.32	2.03**	2.21**	1.48	0.78	0.96
Obese (≥ 30)	1.44	2.30***	3.00**	1.86	1.05	0.89
**Source of regular care**						
Public					1.00	1.00
Mixed					0.79	0.95
Private					0.62*	0.85
**Intercept**	1.95	0.12***	0.60	0.49	5.91*	1.92
**N**	2,175	2,188	1,408	1,405	1,095	1,095

Only selected adjusted odds ratios shown. Complete results for all covariates with standard errors are given in Supplementary Tables. *** *p* <.001, ** *p* <.01, * *p* <.05

Female participants were consistently more likely than males to have been ever screened (aOR 1.92, 95% CI 1.42–2.60), and to be diagnosed (aOR 1.72, 95% CI 1.41–2.11), but once they were diagnosed there were no significant differences between sexes in the likelihood of being treated, or of adherence and control conditional on being treated. Increasing age was associated with increasing likelihood of ever been measured, being diagnosed, and being treated, but was not associated with any statistically significant differences in adherence and control if treated, although control did decrease above the age of 40 years in those being treated.

Household SES quintiles and area SES tertiles were generally not associated with significant differences in the care cascade. However, individuals in the two richest household SES quintiles were more likely to have been ever screened (aORs 1.74 and 1.67 respectively compared with poorest quintile). Additionally, individuals in the second and most developed area SES tertiles were nearly twice as likely as those in the least developed tertile to be treated if diagnosed (aORs 1.93 and 2.13 respectively).

Education and the sector of residence were not associated with any significant differences in progression at each of the cascade steps, and province was not associated with any consistent patterns across the steps.

Increasing body mass index (BMI) was associated with increased likelihood of being diagnosed (aORs 1.66, 2.03, and 2.30 for those of normal weight, overweight and obese respectively versus those who were underweight) and being aware if diagnosed (aORs 1.87, 2.21 and 3.00 for those of normal weight, overweight and obese respectively versus those who were underweight). But differences in BMI were not associated with any significant differences in the likelihood of treatment if diagnosed, or adherence and control if treated.

Individuals with hypertension who self-reported diabetes were three times as likely as those who did not to have been ever screened (aOR 3.14, 95% CI 2.03–4.86) and to be diagnosed (aOR 3.18, 95% CI 2.49–4.04), but were not more likely if diagnosed to be treated, nor more likely to be adherent or be controlled if treated. Receiving care from private providers was negatively associated with adherence and achieving control if being treated, but this was only statistically significant for adherence (aOR 0.62, 95% CI 0.40–0.96).

### Socioeconomic inequalities

A positive concentration index of 0.039 (95% CI 0.024–0.053) for hypertension prevalence indicated that prevalence increased with socioeconomic status after adjusting for age and sex, as expected. The concentration indices for ever screened, diagnosis, awareness, treatment, and compliance conditional on having hypertension were all positive and statistically significant: 0.042 (95% CI 0.019–0.066), 0.048 (95% CI 0.023–0.073), 0.043 (95% CI 0.017–0.069), 0.052 (95% CI 0.024–0.080), and 0.040 (95% CI 0.009–0.071), respectively (Table 4). In contrast, the concentration index for control in those with hypertension, adjusting for age and sex, was 0.003 (95% CI − 0.02–0.026) and not significantly different from zero, indicating no inequality.

**Table 4:** Adjusted concentration indices for attainment of care cascade steps among Sri Lankan adults with hypertension

	**N**	**Concentration index (95% CI)**	*P* value
Hypertension	6,341	0.039 (0.024–0.053)	< 0.001
Ever screened	2,186	0.042 (0.019–0.066)	0.001
Diagnosed	2,175	0.048 (0.023–0.073)	< 0.001
Awareness	2,175	0.043 (0.017–0.069)	0.002
Treated	2,152	0.052 (0.024–0.08)	< 0.001
Adherent	2,126	0.040 (0.009–0.071)	0.012
Controlled	2,152	0.003 (− 0.02 – 0.026)	0.790

A concentration index is a scaled covariance between an outcome and rank in the distribution of socioeconomic status. Adjusted for age and sex. Concentration curves presented in Supplementary Fig. 1. The concentration index for hypertension prevalence is estimated for the full sample, and the others are estimated for those with hypertension

The inequality in treatment was associated with inequalities in use of public and private providers as the source of routine hypertension care (Table 5). Overall, 55% of those receiving hypertension treatment reported they obtained their regular care from public providers, and 37% reported private providers, with the rest using both (8%). Use of public providers alone was pro-poor (concentration index − 0.043), and use of private providers alone was pro-rich (concentration index 0.009). Although use of all care was pro-rich, absence of inequality in control was associated with higher levels of control with public care versus private care.

**Table 5:** Source of regular hypertension care by socioeconomic quintile in Sri Lankan adults with hypertension (%)

	Public (%)	Mixed (%)	Private (%)
Poorest	76.2	9.1	14.7
2	67.2	5.8	27.0
3	61.9	5.8	32.3
4	47.4	8.5	44.1
Wealthiest	31.7	9.2	59.1
Total	55.1	7.8	37.1
Concentration index (95% CI)	− 0.043 (− 0.067 – − 0.018)	0.103 (0.079–0.127)	0.009 (− 0.006 – 0.023)
P value	0.001	< 0.001	0.236

A concentration index is a scaled covariance between an outcome and rank in the distribution of socioeconomic status. Adjusted for age and sex

### International comparison

Shortfalls in the hypertension care cascade are ubiquitous across countries, but benchmarking Sri Lanka against global performance, and regional and health systems peers, can help policymakers to appreciate the significance of gaps, and to identify areas of relative weakness where attention should be prioritized. Unfortunately, it is difficult to make international comparisons owing to lack of consistency between studies in definitions, samples, and methods. In practice, there are only two such multicountry comparisons available. The first is the NCD-RisC modelled estimates of cascade attainment across all countries [1], and the second is a pooled analysis by Geldsetzer et al. of national surveys from 44 LMICs from Europe, Africa, Asia, and Latin America, that used similar definitions [13]. The strength of the NCD-RisC study is that it provides the best available, unbiased, and comparable estimates across all countries, but its limitation are that it relies heavily on imputing estimates even for countries with no data, which limits its value for country-to-country comparisons and also associates its estimates with some degree of uncertainty, and the restriction of its estimates to 30–79 year olds. The strength of the Geldsetzer study is that uses actual survey data from countries, which it then analysed using a consistent protocol ensuring valid comparisons, but its limitation is its limited coverage of countries.

To make best use of these sources, we compared our Sri Lanka results with both studies and then supplemented this by including recent country studies from the South Asia region (geographical peers) and from Malaysia whose health system is most like that of Sri Lanka in terms of structure and disease burden characteristics. We located these other country studies by a literature search and selected the most recent study for each country that used comparable definitions of hypertension prevalence, diagnosis, treatment, and control, adjusting for any differences in the ages covered. This yielded studies from Bangladesh, India and Nepal, but not for Pakistan.

Comparing with South Asian countries, levels of diagnosis, awareness and treatment were substantially higher than in Bangladesh and Nepal, and modestly higher than in India [14, 22–24]. But levels of control in Sri Lankan adults aged ≥ 45 years with hypertension is lower (24%) than reported for Indian adults in the same age range (29–32%), and the disparity is even greater for those with hypertension receiving treatment (45% versus 57–81%) [14, 24]. In the wider region, the care cascade in Sri Lanka in 2019 was very similar to that in Malaysia, an upper-middle income country, in the same year: awareness 50% versus 50%; treatment 44% versus 45%; and control 20% versus 20% respectively [25].

Comparing with results from the pooled analysis of 44 LMICs [13], levels of attainment for blood pressure ever measured, diagnosis, treatment, and control in Sri Lanka (87%, 53%, 44% and 20% respectively) were consistently higher than the pooled averages (74%, 39%, 30% and 10%), falling into the second, second, first and first quintiles of country performance respectively.

To align with the NCD-RisC global estimates for 2019 [1], we re-estimated the care cascade for adults aged 30–79 years old with hypertension in SLHAS sample. Diagnosis, treatment, and control in Sri Lankans were 55%, 46%, and 21% respectively, which was comparable with the NCD-RisC global estimates of 54%, 43% and 21% (averaging the two sexes), lower than average levels in high-income Asia–Pacific countries (69%, 56% and 35%) but higher than the averages for East and South-East Asia (50%, 37%, 15%) and South Asia (38%, 31%, and 14%). Sri Lanka levels of attainment also exhibited greater sex disparity, with the advantage in favour of women being 16%, 14% and 10% for diagnosis, treatment, and control, compared with 10%, 9% and 5% globally.

Although the NCD-RisC estimates indicate performance in the care cascade in Sri Lanka is worse than the average for HICs, performance on diagnosis, treatment and control is better than in some HICs, such as the United Kingdom (UK), where a recent study estimated that only 38% of treated hypertensives aged 40–69 years were controlled [26], compared with 44–49% in Sri Lanka.

## Discussion

Our study is the first to use nationally representative data to estimate the hypertension care cascade in Sri Lanka, and the first to assess inequalities and factors associated with progress along the care cascade. Our principal findings are that only one in two Sri Lankans with hypertension were diagnosed, and only one in five had controlled blood pressure, with the most substantial losses in the cascade occurring at diagnosis (47%) and in achieving control when treated (24%). One additional notable finding is that although most steps in the cascade from screening to treatment were pro-rich, overall control was equitable in relation to socioeconomic status, which contrasts with the usually pro-rich gradient in control in LMICs [13]. That this was associated with higher levels of control in patients receiving public care versus private care also points to the potential importance of effective public sector services in compensating for inequity in access to private care.

In Sri Lanka, women were more likely to be diagnosed, treated, and controlled, which is similar to other countries [1, 13]. In Sri Lanka, this is driven mostly by higher rates in women of ever been measured and of diagnosis. Other than sex differences in health-seeking behaviour, one explanation might be the regular screening of women in Sri Lanka, owing to their interaction with the health system for reproductive and child health care. Young adults are less likely to treated if diagnosed, a pattern that resembles that seen in other countries, but once treated, age disparities in control are less [13]. This disparity at younger ages might be explained by less cumulative interaction with health services which might have led to opportunistic diagnoses, and less aggressive treatment of the young by clinicians.

Whilst we found that sector of residence and province were generally not associated with different outcomes, living in the most developed small areas was associated with higher rates of treatment if diagnosed. This suggests that most geographical inequities in access and service quality occur within regions and maybe related to proximity to specific healthcare providers.

The analyses of household SES yielded contrasting results. The concentration indices showed significant inequality at all steps except control after controlling for age and sex, but the logistic explanatory models that controlled for additional factors did not confirm an independent association with SES, as well as finding no differences between education levels. This suggests that SES inequalities observed are likely mediated by other factors, such as providers used, and being a diabetes patient and BMI, rather than SES influencing demand for or access to care. These findings highlight the need for targeted interventions addressing provider-level determinants, rather than focusing solely on socioeconomic status, to improve care equity.

Provider practices may contribute substantially to losses in the care cascade at the treatment and control steps. This possibility is supported by the findings on the influence of diabetes status, and on inequalities and use of public and private providers. Individuals with known diabetes had higher levels of treatment and control, and this was driven by the frequency of diagnosis being three times higher. This might be explained by standard treatment protocols for diabetes in Sri Lanka, which require frequent monitoring of blood pressure and more aggressive prescribing of antihypertensives. Co-morbidities or provider perception that patients who are overweight or obese may be at greater risk of morbidities may also explain why increased BMI was associated with a higher likelihood of screening and diagnosis.

Although overall treatment was pro-rich, control itself was not inequitable. This was likely due to better levels of control in patients seen by public providers. Whilst this effect was not statistically significant, it appears sufficient to equalize control levels between poorer richer individuals. Such quality-of-care differences would be consistent with previous research that found that public providers performed better than private ones in Sri Lanka on history taking, examination, investigation, and management aspects of quality [27].

These findings underscore the critical role of public sector services in mitigating inequities in health outcomes and highlight the need to strengthen public healthcare provision to sustain this equalizing effect. However, the population-level nature of the data, and the care cascade methodology limits our ability to explain these observed disparities in control and in adherence. Further research is needed that collects data at the level of providers and patients to better understand these dynamics and inform targeted interventions.

International comparisons suggest that Sri Lanka performs relatively well in hypertension control compared to other regional and developing countries, though control rates in treated older adults appear worse than in India [14, 24]. However, this falls short of Sri Lanka’s historical levels of attainment in health which typically approach those of HICs. The comparison with the UK, a laggard in hypertension control amongst HICs, is particularly noteworthy. Despite its predominantly public sector financing and provision of primary care and registration of patients with a single provider – often identified in Sri Lanka as a role model for better primary care, the UK performs worse than Sri Lanka, which relies on mixed public–private financing and provision, and lacks enforced gatekeeping in primary care. This highlights potentially under-appreciated strengths of Sri Lanka’s system, and its similarities with Malaysia’s health system which achieves similar outcomes [25, 28]. To further improve hypertension control, Sri Lanka may benefit more from studying other high performers, such as Canada, the United States and Costa Rica [1].

The COVID- 19 pandemic and a subsequent economic crisis disrupted access to medical services and routine care in Sri Lanka [29], potentially worsening performance across all levels of the care cascade. Nevertheless, the factors influencing the 2019 care cascade performance are likely multifactorial, including clinical inertia [30], and deeply embedded within the health system. Given that changes in care cascade outcomes typically occur over extended periods, and the absence of policymaker appreciation of issues such as clinical inertia and lack of national initiatives to address them, our 2019 estimates may still provide a reasonably accurate profile of the current situation. A follow-up study using new SLHAS data would, however, be critical to update and refine this assessment.

### Strengths and limitations

This study has several limitations, common to most cascade studies. First, we likely overestimated the prevalence of hypertension as blood pressure measurement over multiple visits–as recommended by clinical guidelines–was not feasible within the SLHAS design and resources. Second, as a cross-sectional study, our analysis cannot make causal inferences or assess the temporal relationship between explanatory factors and outcomes. Third, self-reported data on prior blood pressure measurement, routine hypertension care, and treatment may have led to under-estimation of these measures and introduced bias in our logistical models. Fourth, we likely underestimated treatment prevalence by excluding individuals using only non-pharmacological therapies, such as dietary salt restriction, which might also contribute to some of the disparities between population groups. Fifth, voluntary participation and attendance at study field sites could have introduced selection bias if participants were more likely to be diagnosed or compliant, skewing our estimates. Lastly, while the care cascade concept focuses on minimizing the population with uncontrolled hypertension, public health goals should emphasize reducing overall morbidity risk, as blood pressure reductions can be beneficial across all levels of hypertension.

Unlike traditional approaches to the hypertension care cascade, we add ever screened and adherence to medication as additional steps following recent proposals for an extended care cascade [15]. We also distinguish “diagnosed” as a separate step from “awareness,” which is often used interchangeably in the literature, leveraging our data to make this distinction. However, we could not assess the process quality of the treatment that individuals received, as recommended in proposals for an extended care cascade.

A key strength of this study is that it uses data from a large, nationally representative survey covering all adult age groups and districts in Sri Lanka, and which employed trained, dedicated field staff using standard procedures to take clinical measurements. We appropriately accounted for its complex survey design and applied weights in analysis when relevant. Other strengths include the detailed data which were available, such as specifics of medicines used and adherence; sources of regular care; the ability to distinguish awareness from prior diagnosis; and measures of both household and area socioeconomic status. Furthermore, our use of data from a nationally representative longitudinal study provides a foundation for regular tracking of national progress if hypertension is assessed in future study waves.

Further research is still needed to better understand the reasons for shortfalls in hypertension care in Sri Lanka. Our findings point to a substantial failure to achieve control in most treated patients. Quantitative and qualitative studies are needed to explore whether these gaps stem from inadequate blood pressure monitoring, insufficient medication titration to achieve target levels, or other reasons.

## Conclusions

This study provides the first national estimates of the hypertension care cascade in Sri Lanka, establishing a baseline for stakeholders to assess changes in future SLHAS waves. Our findings demonstrate the utility of using the care cascade as a tool to identify performance gaps and potentially to track future national progress.

Among Sri Lankan adults with hypertension, 87% had their blood pressure (BP) ever measured; 53% were diagnosed; 50% were aware; 44% were taking (pharmaceutical) treatment; and only 20% achieved blood pressure control. The largest cascade losses occurred at the steps of diagnosis (47% not diagnosed) and control after treatment (55% of those treated not achieving control). Whilst this finding of substantial losses across the cascade is not surprising, our study is the first to quantify them in Sri Lanka and it should increase awareness of the challenges in translating policy and programs to actual outcomes.

The existence of large cascade losses at different stages is not unique to Sri Lanka, but some countries perform better, indicating the potential for improving performance in Sri Lanka. To develop interventions to improve performance will require understanding the reasons for the shortfalls in performance that our study reveals. Priorities for future research should include understanding why many individuals remain undiagnosed despite frequent healthcare contact and why most treated patients fail to achieve BP control. Mixed-methods research is needed to examine factors and perspectives across the health system, providers, and patients, followed by implementation research to develop effective interventions.

Our findings of large losses in the care cascade at the steps of diagnosis and control, the higher rates of diagnosis in women, and the suggestive evidence that provider differences play an important role, point to two immediate policy goals for hypertension control in Sri Lanka.

The first should be to increase diagnosis rates, particularly in men and younger adults, and the second should be to improve treatment outcomes. The first goal requires increasing detection throughout the whole health system, better exploiting the high rates of medical care utilization in Sri Lanka. Indeed, a recent analysis has shown that an effort to mainstream opportunistic cardiovascular disease screening in routine healthcare encounters in Sri Lanka would be highly cost-effective [31]. The second goal requires urgent and concerted efforts to understand the reasons why physicians fail to achieve blood pressure control targets, and then to implement interventions to improve physician practices throughout the healthcare system, including in the private sector, and possibly to adopt better treatment strategies. The latter might consider shifting standard therapies to combination therapies (polypill) and increased use of technology in risk assessment and care management, which have demonstrated improvements in hypertension control in clinical trial studies in both Sri Lanka and Malaysia [32, 33].

Improving hypertension outcomes in Sri Lanka will not be easy as the shortfalls in coverage we found occur despite significant national efforts to improve hypertension care. Our results point to ways in which national strategy can be refocused, but they also point to a need to consider the experience of a wider range of global exemplars and clinical trials evidence to identify practical solutions to guide national policy.

## Supplementary Information

Supplementary Material 1

## Data Availability

The SLHAS has adopted an Open Data policy. It will make Wave 1 data available to other qualifying researchers on application. Interested researchers may contact the SLHAS at slhas @ ihp.lk.

## Abbreviations

ACC/AHA: American College of Cardiology/American Heart Association
aOR: Adjusted odds ratio
ASES: Area socioeconomic status
ATC: Anatomical Therapeutic Chemical
BMI: Body mass index
BP: Blood pressure
CI: Confidence interval
DBP: Diastolic blood pressure
GND: Grama niladhari division
HIC: High-income country
IPF: Iterative proportional fitting
JNC: Joint National Committee
LMIC: Low and middle-income country
MOH: Ministry of Health
PCA: Principal components analysis
PSU: Primary sampling unit
SES: Socioeconomic status
SLHAS: Sri Lanka Health and Ageing Study
SBP: Systolic blood pressure
UK: United Kingdom
WHO: World Health Organization
