# Supplementary material for: Hypertension diagnosis, awareness, treatment, and control in Sri Lankan adults: a nationally representative cross-sectional study
Source: BMC Public Health. 2025 Apr 24;25:1531. doi: 10.1186/s12889-025-22659-7 (PMC12020379; doi:10.1186/s12889-025-22659-7)
Supplement: Supplementary file 1 — Supplementary Material 1 [file 12889_2025_22659_MOESM1_ESM.docx]

# Supplementary Materials

Supplementary Table 1: Sample characteristics

|  | **All study participants (N=6,342)** | | |  | **Participants with hypertension (N=2,218)** | | |
| --- | --- | --- | --- | --- | --- | --- | --- |
| **Characteristic** | **N** | **Unweighted percentage (%)** | **Weighted percentage (%)** |  | **N** | **Unweighted percentage (%)** | **Weighted percentage (%)** |
| **Hypertension** | | | | | | | |
| No | 4,124 | 65.0 | 72.4 |  |  |  |  |
| Yes | 2,218 | 35.0 | 27.6 |  | 2,218 | 100.0 | 100.0 |
| **Sex** | | | | | | | |
| Male | 3,107 | 49.0 | 47.6 |  | 1,095 | 49.4 | 46.9 |
| Female | 3,235 | 51.0 | 52.4 |  | 1,123 | 50.6 | 53.1 |
| **Age (years)** | | | | | | | |
| 18-29 | 932 | 14.7 | 25.1 |  | 64 | 2.9 | 5.8 |
| 30-39 | 1,088 | 17.2 | 20.8 |  | 157 | 7.1 | 10.9 |
| 40-49 | 1,126 | 17.8 | 18.0 |  | 286 | 12.9 | 17.4 |
| 50-59 | 1,101 | 17.4 | 16.2 |  | 452 | 20.4 | 22.9 |
| 60-69 | 1,099 | 17.3 | 11.6 |  | 593 | 26.7 | 23.1 |
| 70-79 | 821 | 12.9 | 6.1 |  | 550 | 24.8 | 14.8 |
| 80+ | 175 | 2.8 | 2.1 |  | 116 | 5.2 | 5.0 |
| **Ethnicity** | | | | | | | |
| Sinhala | 4,466 | 70.4 | 75.2 |  | 1,534 | 69.2 | 73.5 |
| SL Tamil | 1,264 | 19.9 | 12.6 |  | 457 | 20.6 | 11.8 |
| Indian Tamil | 201 | 3.2 | 2.7 |  | 76 | 3.4 | 3.2 |
| Muslim | 411 | 6.5 | 9.5 |  | 151 | 6.8 | 11.5 |
| **Sector** | | | | | | | |
| Urban | 1,923 | 30.3 | 19.6 |  | 784 | 35.3 | 24.4 |
| Rural | 3,483 | 54.9 | 70.6 |  | 1,108 | 50.0 | 66.7 |
| Estate | 168 | 2.6 | 0.7 |  | 70 | 3.2 | 0.8 |
| Rural/Estate | 768 | 12.1 | 9.2 |  | 256 | 11.5 | 8.0 |
| **Household SES quintile** | | | | | | | |
| Poorest | 1,506 | 23.7 | 20.0 |  | 502 | 22.6 | 18.9 |
| 2 | 1,266 | 20.0 | 20.0 |  | 433 | 19.5 | 18.3 |
| 3 | 1,170 | 18.4 | 20.0 |  | 390 | 17.6 | 19.6 |
| 4 | 1,156 | 18.2 | 20.0 |  | 423 | 19.1 | 21.6 |
| Wealthiest | 1,244 | 19.6 | 20.0 |  | 470 | 21.2 | 21.7 |
| **Diabetes (self-reported)** | | | | | | | |
| No | 5,283 | 83.3 | 86.1 |  | 1,594 | 71.9 | 73.7 |
| Yes | 1,057 | 16.7 | 13.9 |  | 624 | 28.1 | 26.3 |
| **Area SES tertile** | | | | | | | |
| Least developed | 2,313 | 36.5 | 33.1 |  | 706 | 31.8 | 28.5 |
| 2 | 1,807 | 28.5 | 33.1 |  | 589 | 26.6 | 30.6 |
| Most developed | 2,222 | 35.0 | 33.8 |  | 923 | 41.6 | 40.9 |
| **Province** | | | | | | | |
| Western | 1,338 | 21.1 | 30.0 |  | 534 | 24.1 | 35.8 |
| Central | 929 | 14.6 | 12.5 |  | 377 | 17.0 | 14.0 |
| Southern | 812 | 12.8 | 12.3 |  | 256 | 11.5 | 10.5 |
| Northern | 678 | 10.7 | 5.0 |  | 232 | 10.5 | 4.5 |
| Eastern | 534 | 8.4 | 6.8 |  | 155 | 7.0 | 4.8 |
| North-Western | 515 | 8.1 | 11.7 |  | 162 | 7.3 | 10.8 |
| North-Central | 459 | 7.2 | 6.1 |  | 130 | 5.9 | 4.7 |
| Uva | 452 | 7.1 | 6.1 |  | 151 | 6.8 | 5.5 |
| Sabaragamuwa | 625 | 9.9 | 9.7 |  | 221 | 10.0 | 9.5 |

*Notes:* In the sector categorization, rural/estate refers to primary sampling units that were a mix of rural and estate sectors. Population reference values taken from the 2012 national census statistics adjusted for demographic change during 2012–2019.

Supplementary Table 2: Adjusted odds ratios (and standard errors) for logistic regression models of being ever screened or diagnosed if having hypertension in Sri Lankan adults (≥18 years), SLHAS 2018/2019 estimates.

|  | **Ever screened** | **Diagnosed** |
| --- | --- | --- |
| **Sex** |  |  |
| Male | 1.00 | 1.00 |
| Female | 1.92*** (1.42–2.60) | 1.72*** (1.41–2.11) |
|  |  |  |
| **Age (years)** |  |  |
| 18-29 | 0.47* (0.24–0.92) | 0.03** (0.00–0.26) |
| 30-39 | 0.63 (0.38–1.05) | 0.45** (0.28–0.73) |
| 40-49 | 1.00 | 1.00 |
| 50-59 | 1.50 (0.95–2.37) | 2.27*** (1.64–3.14) |
| 60-69 | 1.96** (1.25–3.07) | 3.14*** (2.29–4.32) |
| 70-79 | 2.54*** (1.56–4.13) | 6.71*** (4.74–9.49) |
| 80+ | 4.81** (1.78–12.97) | 7.84*** (4.53–13.56) |
|  |  |  |
| **Sector** |  |  |
| Urban | 1.00 | 1.00 |
| Rural | 1.08 (0.71–1.65) | 0.90 (0.68–1.19) |
| Estate | 1.50 (0.62–3.61) | 1.49 (0.73–3.06) |
| Rural/Estate | 1.33 (0.72–2.46) | 0.80 (0.51–1.24) |
|  |  |  |
| **Ethnicity** |  |  |
| Sinhala | 1.00 | 1.00 |
| SL Tamil | 0.41*** (0.25–0.70) | 0.89 (0.59–1.32) |
| Indian Tamil | 0.22*** (0.11–0.42) | 0.97 (0.55–1.72) |
| Muslim | 0.49* (0.26–0.91) | 1.59 (1.00–2.52) |
|  |  |  |
| **Education** |  |  |
| No schooling | 1.00 | 1.00 |
| Primary | 1.17 (0.62–2.21) | 1.05 (0.65–1.70) |
| Secondary | 1.39 (0.75–2.57) | 1.24 (0.78–1.98) |
| Tertiary | 3.70* (1.08–12.61) | 1.02 (0.50–2.10) |
|  |  |  |
| **Household SES quintile** |  |  |
| Poorest | 1.00 | 1.00 |
| 2 | 0.97 (0.65–1.45) | 1.19 (0.87–1.61) |
| 3 | 1.48 (0.92–2.38) | 1.06 (0.76–1.48) |
| 4 | 1.74* (1.05–2.86) | 1.12 (0.80–1.57) |
| Wealthiest | 1.67* (1.00–2.77) | 1.10 (0.77–1.55) |
|  |  |  |
| **Area SES tertile** |  |  |
| Least developed | 1.00 | 1.00 |
| 2 | 1.20 (0.78–1.85) | 0.99 (0.74–1.34) |
| Most developed | 0.83 (0.50–1.38) | 1.11 (0.77–1.60) |
|  |  |  |
| **Diabetes (self-reported)** | 3.14*** (2.03–4.86) | 3.18*** (2.49–4.04) |
|  |  |  |
| **WHO BMI categories** |  |  |
| Underweight (<18.5) | 1.00 | 1.00 |
| Normal (18.5-24.9) | 1.04 (0.61–1.78) | 1.66* (1.12–2.45) |
| Overweight (25–29.9) | 1.32 (0.74–2.38) | 2.03** (1.32–3.11) |
| Obese (≥30) | 1.44 (0.72–2.85) | 2.30*** (1.40–3.76) |
|  |  |  |
| **Province** |  |  |
| Western | 1.00 | 1.00 |
| Central | 1.10 (0.64–1.91) | 1.23 (0.86–1.76) |
| Southern | 0.82 (0.48–1.40) | 1.05 (0.74–1.50) |
| Northern | 1.56 (0.75–3.24) | 1.54 (0.89–2.67) |
| Eastern | 1.84 (0.88–3.83) | 1.06 (0.63–1.76) |
| North-Western | 1.25 (0.61–2.57) | 1.55 (0.99–2.43) |
| North-Central | 0.60 (0.31–1.15) | 1.69* (1.04–2.72) |
| Uva | 0.77 (0.42–1.42) | 1.31 (0.83–2.05) |
| Sabaragamuwa | 0.66 (0.37–1.17) | 1.25 (0.84–1.85) |
|  |  |  |
| **Intercept** | 1.95 (0.67–5.69) | 0.12*** (0.05–0.25) |

*Notes:* *** p<.001, ** p<.01, * p<.05

Supplementary Table 3: Adjusted odds ratios (and standard errors) for logistic regression models of being aware or treated if diagnosed with hypertension in Sri Lankan adults (≥18 years), SLHAS 2018/2019 estimates.

|  | **Aware** | **Treated** |
| --- | --- | --- |
| **Sex** |  |  |
| Male | 1.00 | 1.00 |
| Female | 1.04 (0.77–1.41) | 0.91 (0.69–1.21) |
|  |  |  |
| **Age (years)** |  |  |
| 18-29 | 0.10 (0.01–1.25) | 1.00 |
| 30-39 | 0.95 (0.36–2.50) | 0.61 (0.28–1.34) |
| 40-49 | 1.00 | 1.00 |
| 50-59 | 1.32 (0.74–2.34) | 1.85* (1.14–3.01) |
| 60-69 | 1.09 (0.63–1.86) | 2.36*** (1.48–3.77) |
| 70-79 | 1.77* (1.00–3.12) | 3.03*** (1.86–4.92) |
| 80+ | 1.74 (0.81–3.73) | 3.64*** (1.79–7.42) |
|  |  |  |
| **Sector** |  |  |
| Urban | 1.00 | 1.00 |
| Rural | 1.46 (0.96–2.24) | 0.89 (0.60–1.32) |
| Estate | 1.43 (0.43–4.78) | 1.37 (0.52–3.60) |
| Rural/Estate | 0.90 (0.46–1.73) | 1.36 (0.73–2.56) |
|  |  |  |
| **Ethnicity** |  |  |
| Sinhala | 1.00 | 1.00 |
| SL Tamil | 1.04 (0.58–1.85) | 0.79 (0.47–1.36) |
| Indian Tamil | 2.30 (0.75–7.05) | 0.50 (0.24–1.07) |
| Muslim | 1.31 (0.67–2.59) | 0.61 (0.36–1.06) |
|  |  |  |
| **Education** |  |  |
| No schooling | 1.00 | 1.00 |
| Primary | 1.00 (0.52–1.94) | 1.14 (0.64–2.04) |
| Secondary | 1.40 (0.74–2.64) | 1.40 (0.81–2.44) |
| Tertiary | 1.33 (0.45–3.99) | 0.56 (0.23–1.36) |
|  |  |  |
| **Household SES quintile** |  |  |
| Poorest | 1.00 | 1.00 |
| 2 | 1.09 (0.68–1.74) | 0.94 (0.62–1.42) |
| 3 | 0.82 (0.51–1.33) | 0.78 (0.50–1.20) |
| 4 | 1.09 (0.66–1.82) | 1.53 (0.95–2.48) |
| Wealthiest | 0.93 (0.56–1.56) | 1.21 (0.75–1.95) |
|  |  |  |
| **Area SES tertile** |  |  |
| Least developed | 1.00 | 1.00 |
| 2 | 1.46 (0.93–2.29) | 1.93** (1.29–2.89) |
| Most developed | 1.68 (0.95–2.95) | 2.13** (1.29–3.53) |
|  |  |  |
| **Diabetes (self-reported)** | 1.02 (0.75–1.40) | 1.10 (0.82–1.47) |
|  |  |  |
| **WHO BMI categories** |  |  |
| Underweight (<18.5) | 1.00 | 1.00 |
| Normal (18.5-24.9) | 1.87* (1.07–3.26) | 1.25 (0.72–2.17) |
| Overweight (25–29.9) | 2.21** (1.21–4.02) | 1.48 (0.82–2.66) |
| Obese (≥30) | 3.00** (1.43–6.29) | 1.86 (0.94–3.70) |
|  |  |  |
| **Province** |  |  |
| Western | 1.00 | 1.00 |
| Central | 2.16** (1.24–3.76) | 1.38 (0.85–2.25) |
| Southern | 1.29 (0.77–2.14) | 1.22 (0.74–2.02) |
| Northern | 1.60 (0.73–3.51) | 2.65* (1.25–5.61) |
| Eastern | 1.36 (0.64–2.88) | 1.11 (0.58–2.13) |
| North-Western | 2.11* (1.03–4.30) | 1.37 (0.76–2.47) |
| North-Central | 1.33 (0.70–2.55) | 1.18 (0.65–2.17) |
| Uva | 1.68 (0.87–3.25) | 1.98* (1.03–3.80) |
| Sabaragamuwa | 1.59 (0.89–2.82) | 1.12 (0.65–1.91) |
|  |  |  |
| **Intercept** | 0.60 (0.18–1.98) | 0.49 (0.16–1.45) |

*Notes:* *** p<.001, ** p<.01, * p<.05

Supplementary Table 4: Adjusted odds ratios (and standard errors) for logistic regression models of being adherent or controlled if being treated for hypertension in Sri Lankan adults (≥18 years), SLHAS 2018/2019 estimates.

|  | **Adherent** | **Controlled** |
| --- | --- | --- |
| **Sex** |  |  |
| Male | 1.00 | 1.00 |
| Female | 0.86 (0.58–1.27) | 1.23 (0.95–1.61) |
|  |  |  |
| **Age (years)** |  |  |
| 30-39 | 0.85 (0.23–3.13) | 0.72 (0.24–2.13) |
| 40-49 | 1.00 | 1.00 |
| 50-59 | 1.59 (0.78–3.20) | 0.83 (0.49–1.43) |
| 60-69 | 1.42 (0.73–2.76) | 0.81 (0.48–1.37) |
| 70-79 | 2.03* (1.01–4.07) | 0.60 (0.35–1.01) |
| 80+ | 2.69 (0.94–7.71) | 0.74 (0.38–1.44) |
|  |  |  |
| **Sector** |  |  |
| Urban | 1.00 | 1.00 |
| Rural | 1.33 (0.80–2.23) | 0.98 (0.69–1.39) |
| Estate | 1.54 (0.44–5.40) | 1.05 (0.36–3.00) |
| Rural/Estate | 1.74 (0.70–4.34) | 0.93 (0.52–1.66) |
|  |  |  |
| **Ethnicity** |  |  |
| Sinhala | 1.00 | 1.00 |
| SL Tamil | 0.36** (0.18–0.73) | 0.50* (0.29–0.86) |
| Indian Tamil | 0.42 (0.14–1.28) | 0.27** (0.11–0.71) |
| Muslim | 0.48* (0.24–0.99) | 0.27*** (0.14–0.50) |
|  |  |  |
| **Education** |  |  |
| No schooling | 1.00 | 1.00 |
| Primary | 0.59 (0.21–1.69) | 0.92 (0.50–1.71) |
| Secondary | 0.61 (0.22–1.66) | 0.67 (0.37–1.21) |
| Tertiary | 0.57 (0.13–2.57) | 0.58 (0.22–1.55) |
|  |  |  |
| **Household SES quintile** |  |  |
| Poorest | 1.00 | 1.00 |
| 2 | 1.00 (0.54–1.84) | 0.83 (0.55–1.25) |
| 3 | 0.87 (0.46–1.63) | 0.96 (0.62–1.48) |
| 4 | 0.89 (0.48–1.67) | 1.08 (0.70–1.64) |
| Wealthiest | 1.60 (0.80–3.21) | 0.94 (0.61–1.47) |
|  |  |  |
| **Area SES tertile** |  |  |
| Least developed | 1.00 | 1.00 |
| Middle | 0.77 (0.42–1.39) | 0.93 (0.63–1.38) |
| Most developed | 1.43 (0.68–3.00) | 0.86 (0.54–1.39) |
|  |  |  |
| **Diabetes (self-reported)** | 1.19 (0.80–1.76) | 0.91 (0.70–1.18) |
|  |  |  |
| **WHO BMI categories** |  |  |
| Underweight (<18.5) | 1.00 | 1.00 |
| Normal (18.5-24.9) | 0.95 (0.37–2.41) | 1.37 (0.79–2.39) |
| Overweight (25–29.9) | 0.78 (0.30–2.05) | 0.96 (0.54–1.73) |
| Obese (≥30) | 1.05 (0.36–3.02) | 0.89 (0.46–1.72) |
|  |  |  |
| **Province** |  |  |
| Western | 1.00 | 1.00 |
| Central | 1.08 (0.57–2.05) | 0.65 (0.41–1.03) |
| Southern | 2.70* (1.24–5.89) | 0.89 (0.57–1.38) |
| Northern | 2.07 (0.84–5.09) | 2.25* (1.13–4.50) |
| Eastern | 1.77 (0.72–4.36) | 2.15* (1.09–4.24) |
| North-Western | 1.59 (0.71–3.60) | 1.22 (0.71–2.12) |
| North-Central | 2.62 (0.93–7.36) | 0.80 (0.45–1.43) |
| Uva | 4.13* (1.16–14.69) | 0.84 (0.47–1.50) |
| Sabaragamuwa | 2.14 (0.93–4.91) | 1.20 (0.72–1.98) |
|  |  |  |
| **Source of regular care** |  |  |
| Public | 1.00 | 1.00 |
| Mixed | 0.79 (0.39–1.57) | 0.95 (0.59–1.53) |
| Private | 0.62* (0.40–0.96) | 0.85 (0.63–1.15) |
|  |  |  |
| Intercept | 5.91* (1.06–32.96) | 1.92 (0.63–5.86) |

*Notes:* *** p<.001, ** p<.01, * p<.05

Supplementary Figure 1. Concentration curves for hypertension prevalence, ever screened, diagnosed, awareness, treatment, adherent, and controlled, Sri Lankan adults (age≥18 years), Sri Lanka Health and Ageing Study 2018/2019 estimates.

| 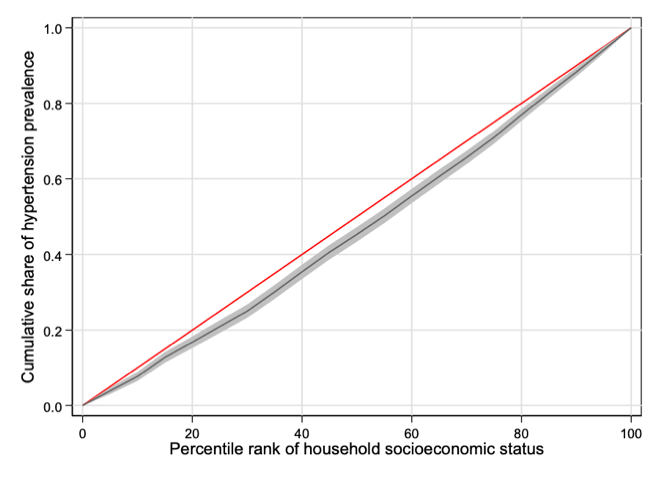 | 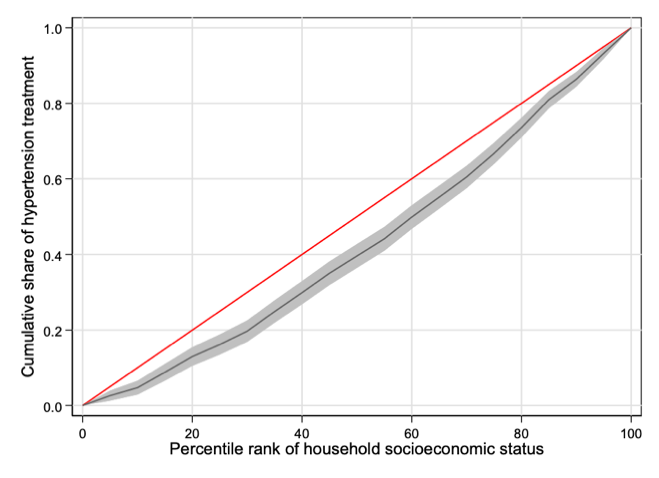 |
| --- | --- |
| 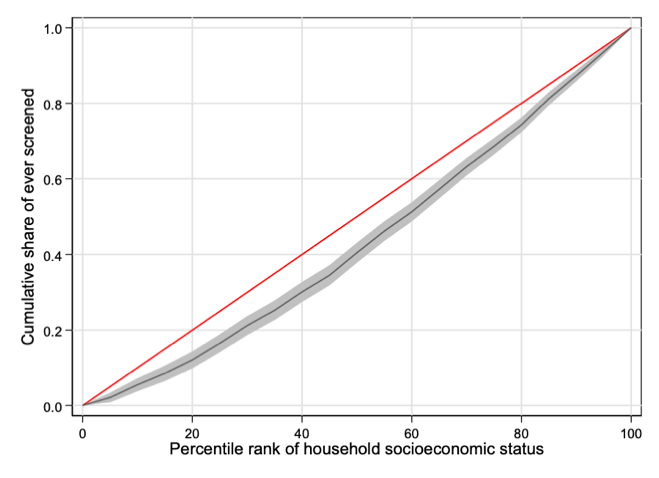 | 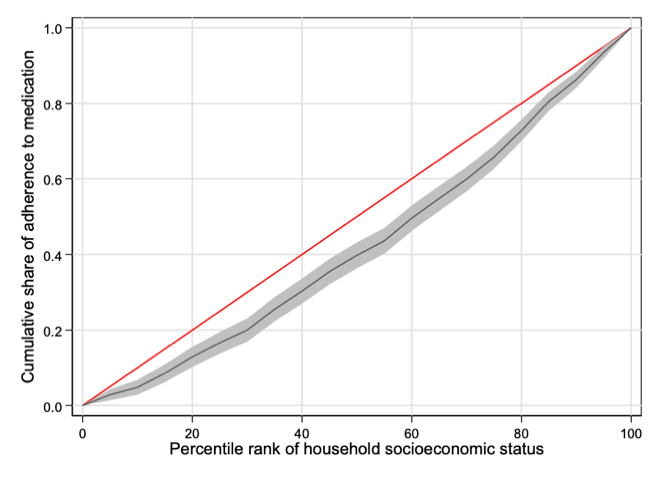 |
| 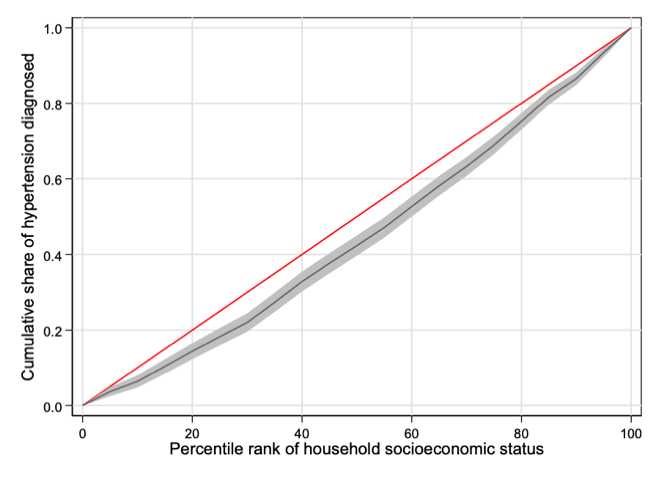 | 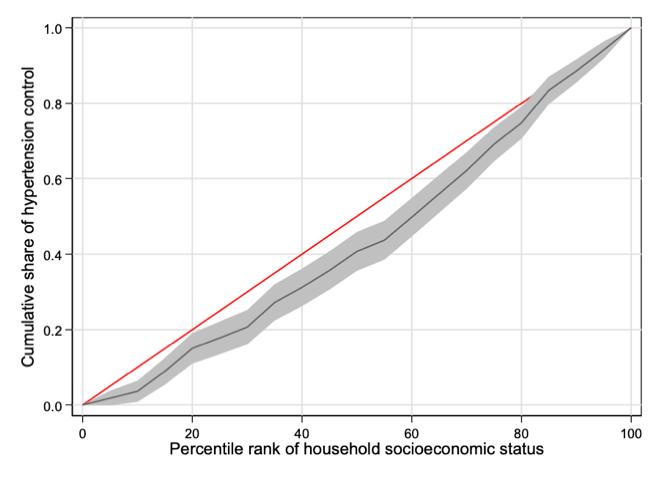 |
| 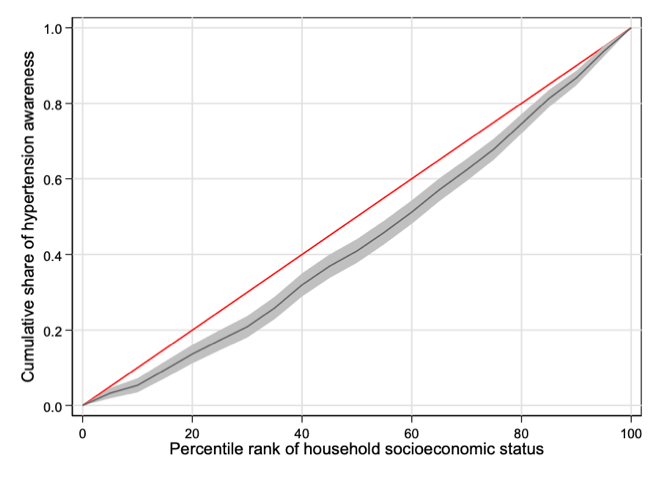 |  |
